# Expression and distribution of Toll-like receptors 11–13 in the brain during murine neurocysticercosis

**DOI:** 10.1186/1742-2094-5-53

**Published:** 2008-12-12

**Authors:** Bibhuti B Mishra, Uma Mahesh Gundra, Judy M Teale

**Affiliations:** 1 Department of Biology, South Texas Center for Emerging Infectious Diseases, The University of Texas at San Antonio, One UTSA Circle, San Antonio, Texas 78249-1644, USA

## Abstract

The functions of Toll-like receptors (TLRs) 11–13 in central nervous system (CNS) infections are currently unknown. Using a murine model of neurocysticercosis, we investigated the expression and distribution of TLRs 11–13 by using both gene specific real-time PCR analysis and *in situ *immunofluoresence microscopy in both control and neurocysticercosis brains. In the mock infected brain, mRNAs of TLRs 11–13 were constitutively expressed. Parasite infection caused an increase of both mRNAs and protein levels of all three TLRs by several fold. All three TLR proteins were present in both CNS and immune cell types. Among them TLR13 was expressed the most in terms of number of positive cells and brain areas expressing it, followed by TLR11 and TLR12 respectively. Among the nervous tissue cells, TLRs 11–13 protein levels appeared highest in neurons. However, TLR13 expression was also present in ependymal cells, endothelial cells of pial blood vessels, and astrocytes. In contrast, infiltrating CD11b and CD11c positive myeloid cells predominantly produced TLR11 protein, particularly early during infection at 1 wk post infection (~50% cells). TLRs 12 and 13 proteins were present on approximately 5% of infiltrating immune cells. The infiltrating cells positive for TLRs 11–13 were mostly of myeloid origin, CD11b+ cells. This report provides a comprehensive analysis of the expression of TLRs 11–13 in normal and parasite infected mouse brains and suggests a role for them in CNS infections.

## Background

Neurocysticercosis (NCC) is the most common parasitic disease of the central nervous system (CNS) caused by the larvae of *Taenia solium *[1]. This disease is a public health problem in many third world and developing countries [1-3]. The symptomatic phase of the disease includes clinical signs such as epilepsy [2], increased intracranial (i.c.) pressure, obstructive hydroencephalus, stroke, and encephalitis [1,4]. Autopsy specimens of symptomatic patients reveal evidence of inflammation consisting of a chronic granulomatous reaction [4]. The observed inflammation probably is detrimental. Considering the CNS is devoid of a classically defined lymphatic system, the innate immune response might play a major role in this process.

Toll-like receptors are key host molecules in innate immune responses during infections [5]. To date, thirteen mammalian TLR paralogues have been identified (10 in humans and 12 in mice) [6]. These receptors are highly conserved proteins that recognize distinct mutation resistant molecular patterns common to pathogens, termed pathogen-associated molecular patterns (PAMPs) [7,8]. Ligand recognition by TLRs culminates invariably in the manifestation of inflammatory responses and induction of adaptive immune responses [9,10]. Growing evidence suggests that TLRs 2, 3, 4, and 9 participate in host immune responses in a variety of CNS diseases [11-23]. Furthermore, recent studies have shown that several nervous tissue cells upregulate particular TLRs as a result of infection, trauma, or autoimmune disease [24-29]. However, little information is available for TLR functions in NCC; indeed, the expression profile of TLRs 11–13 is unknown in any CNS disease.

In an experimental murine model for NCC, mice receive intracranial (i.c.) inoculations of *Mesocestoides corti *(*M. corti*) metacestodes. The brain immune response in this model is associated with a predominant TH1 pathway of cytokine responses [30,31]. Analysis of the expressions and distributions of TLRs 1–9 suggested that *M. corti *parasite infection increased both gene expression and protein levels of each TLRs1–9 several fold except TLR5 where only the mRNA was upregulated [12]. In addition, these TLRs were differentially distributed among various CNS cell types and infiltrating leukocytes. In this study, we preformed gene specific Real-time polymerase chain reaction (RT-PCR) analysis to detect TLRs 11–13 at the mRNA level for both infected and mock-infected mice. *In situ *immunofluoresence (IF) microscopy, using antibodies specific for each of the TLRs in combination with antibodies for distinct cell surface markers, determined the expression of TLRs by particular cell types in infected and uninfected brains. The data obtained from these two approaches implicate TLRs 11–13 in host immune surveillance in the CNS, particularly in NCC.

## Methods

### Mice

Female Balb/c mice used in this study were purchased from the National Cancer Institute Animal Program (Bethesda, MD). Experiments were conducted under the guidelines of the IACUC, UTSA, University of Texas System, the US Department of Agriculture, and the National Institutes of Health.

### Murine model of neurocysticercosis

In this study we used a well characterized mouse model of NCC developed in our laboratory [30,32]. Briefly, we maintained larvae of *M. corti *parasites by serial, intraperitoneal (i.p.) inoculations of 6–8 wk old female Balb/c mice. We harvested the larvae aseptically and induced murine NCC by intracranial injections of 50 μl of Hank's Buffered Salt Solution (HBSS) containing approximately 40 parasites into 3–5 week old mice under short term anesthesia. Mock infected control mice were injected by the intracranial route with 50 μl sterile HBSS using the same protocol.

### RNA isolation and real-time PCR analysis

To determine the CNS gene expression of TLRs 11–13 in murine NCC, brains were removed from infected and mock infected control mice at 1, 3, 6, and 10 wk post infection (p.i.) (n = 3 in each group). Animals were perfused prior to sacrifice to avoid RNA contamination from blood cells. Briefly, mice were anaesthetized with 100 μl of mouse cocktail containing 100 mg/ml of ketamine and 20 mg/ml of rompum (Laboratory animal resource, UTHSCSA, TX) and perfused with 10 ml of PBS, pH 7.4 through the left ventricle. Brains were immediately removed after perfusion and total RNA was extracted using Trizol reagent (Invitrogen) according to manufacturers' instructions. Real-time PCR analysis of these samples, using SYBR green as the detection dye, measured the expression levels of TLR-specific mRNAs. One microgram of total RNA from either infected or mock infected mice was reverse transcribed into cDNA by using a high capacity cDNA reverse transcription kit according to the manufacturers' instructions (Applied Biosystems, CA, USA). Transcript levels of the housekeeping ribosomal 18S and TLRs 11–13 were PCR amplified in each sample by using specific primers (Advanced Nucleic Acids Core Facility, UTHSCSA, TX). Sequences of the specific primers used for 18S and TLRs 11–13 are as follows: 18S (sense) 5'-CATGTGGTGTTGAGGAAAGCA-3' and (anti sense) 5'-GTCGTGGGTTCTGCATGATG-3'; TLR11 (sense) 5'-TCCTTCCTCTGATTAGCTGTCCTAA-3' and (antisense) 5'-TCCACATAATTTCCACCAACAAGT-3'; TLR12 (sense) 5'-GCCGCCATTCCAAGCTA TC-3' and (antisense) 5'-CTCCACAGTCCGAGGTACAACTT-3'; and TLR13 (sense) 5'-ATGGCACAAAACGGAGAAGAA-3' and (antisense) 5'-CTTTGTATACCCATGCCTCATCAG-3'. The target expression levels were normalized to levels of the house keeping 18S in the same sample. The normalized values give the relative abundance of mRNA for each TLR gene.

### Antibodies

Anti-mouse antibodies were purchased from the following companies: TLR11 from Serotec (Raleigh, NC), TLRs 12–13 from Imgenex (San Diego, CA.), biotinylated or R-Phycoerythrin (PE) conjugated CD11b [myeloid cells] from Pharmingen (San Diego, CA.), and Biotinylated Neuronal nuclear protein [Neun] from US Biological (Swampscott, MA.). Anti-mouse glial fibrillary protein [GFAP] (Pharmingen) was conjugated to Alexa Fluor 488 (Molecular Probes) according to manufacturers' instructions. For indirect immunofluoresence, rabbit anti-TLRs 11, 12, and 13 followed by Alexa 488 conjugated (green) chicken anti-rabbit (Molecular Probes) or Rhodamine red-conjugated (red) goat anti-rabbit secondary antibodies (Jackson laboratory) were used. Biotinylated primary antibodies were detected using Alexa Fluor 488-labeled streptavidin (Molecular Probes).

### Immunofluorescence

We used *in situ *immunofluorescence (IF) to detect tissue expression and distribution of TLRs in the CNS. Parasite infected brains were analyzed at various times post infection (1 wk, 3 wk, and 6 wk) and compared to mock-infected brains from control animals. Brains were immediately removed after perfusion (as described above), embedded in optimal cutting temperature (OCT) medium, and snap frozen as previously described [12,33]. Serial horizontal cryosections, 10 μm in thickness, were placed on silane prep slides (Sigma Biosciences, St. Louis, Mo). One in every five slides was fixed in formalin for 12 min at room temperature and stained with hematoxylin and eosin. The remaining slides were air dried overnight and fixed in fresh acetone for 20s at room temperature. Acetone-fixed sections were wrapped in aluminum foil and stored at -80°C or processed immediately for IF.

IF analysis determined the location of individual TLRs in brain sections. All steps were carried out at room temperature. Sections were incubated with TLR-specific primary antibodies in PBS buffer with 3% host serum to prevent non-specific binding. After 1 hr, sections were washed seven times for 3 min each in 50 mM Tris-HCl, pH 7.6 with 0.1% Tween-20 and incubated with appropriate secondary antibodies for 30 min. Sections were then washed seven times 3 min each in 50 mM Tris-HCl, pH 7.6 with 0.1% Tween-20. The above mentioned procedures were sequentially repeated for double IF staining. The sections were mounted using FluorSave reagent (Calbiochem, La Jolla, CA) containing 0.3 μM 4', 6'-diamidino-2-phenylidole (DAPI)-diacetate (Molecular Probes). Additional control staining was performed to rule out any nonspecific staining. In each case, sections were blocked with saturating concentrations of appropriate host serum antibodies to eliminate false positive staining due to FcR-mediated nonspecific binding. Staining in the absence of primary antibodies provided additional negative controls.

### Quantification of TLRs 11–13 protein expression

Sections of brains from mock-infected and NCC mice were stained with specific anti-TLR antibodies followed by labeled secondary antibodies to quantify the differences in TLR 11–13 protein levels. Images at 20× magnification of a number of brain areas from the entire brain of each mock infected and NCC animal were taken using identical camera settings so that the number and intensity of pixels would reflect the differences in their protein expression. The atlas of the mouse brain and spinal cord book by Sidman RL [34] provided neuroanatomy guidance to select identical brain areas for analysis. Protein level of a particular TLR was determined by measuring the area (number of pixels) and fluorescent intensity (average intensity of pixels) of the staining from twenty four images captured randomly in the CNS parenchyma from each mock and parasite infected brain exhibiting TLR protein staining on nervous tissue cells. The unit area of each image was 0.172 sq. mm. Analysis was performed by using the IP Lab 4.0 imaging software (BD Biosciences Bioimaging, Rockville MD). The relative TLR protein expression was calculated by multiplying the number of pixels (area) by the average intensity of pixels.

### Statistical analysis

We used the Student's *t*-test for comparison of means of different groups [SIGMA PLOT 8.0 (Systat Software, San Jose, CA)]. A *P *value less than 0.05 was considered to be statistically significant.

## Results

### mRNA expression of TLRs 11–13 in normal and infected brain tissue

**Figure 1:**
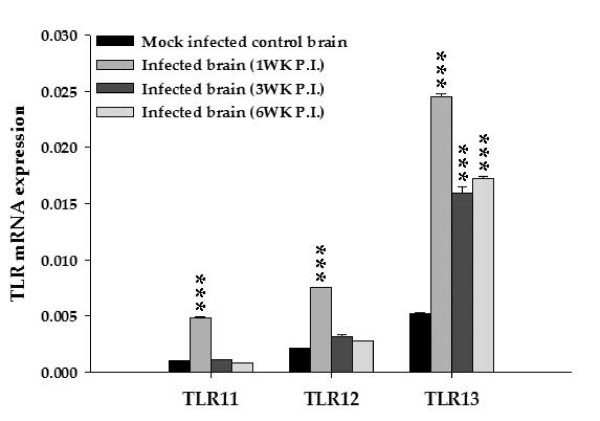
***M. corti *infection elevates mRNA expression of TLRs 11–13 in brain**. Total RNA was isolated from infected and mock infected control mouse brains at 1 wk, 3 wk, and 6 wk p.i. using Trizol reagent. Isolated RNA was reversed transcribed to cDNA by using random primers. Levels of TLR specific RNA and housekeeping gene 18S in these samples were measured by Real Time PCR analysis as described below using SYBR green as the detection dye. TLR specific mRNA levels were normalized to the mRNA level of the housekeeping gene 18S in the same sample and expressed in arbitrary units. The normalized values corresponding to the mRNA expression for each TLR gene are shown in the y-axis and represent the mean of three independent experiments. The data obtained were compared using a Student's *t*-test.

Quantitaive real-time PCR analysis was performed to determine the expression profile of TLRs 11–13 in mock infected and parasite infected brain. Constitutive mRNA expression was present for each TLR with a relatively higher level of TLR13 gene expression. (Fig. 1). Upon infection, gene expression was significantly upregulated at 1 wk p.i. (P < 0.001). At 3 wk p.i., mRNAs of TLR11 and TLR12 almost returned to normal baseline levels and remained so at 6 wk p.i. (Fig. 1) and 10 wk p.i. (data not shown). In contrast, gene expression of TLR13 was significantly upregulated at all the post infection times tested. This upregulation was greatest at 1 wk p.i. with a relatively lower level at later stages (3 wk, 6 wk) (Fig. 1) and 10 wk (data not shown). Among TLRs 11–13, expression of TLR13 was the highest in both normal and parasitic infected brains and remained at an elevated level throughout infection (Fig. 1). These results indicate that TLR 11–13 mRNAs are expressed in normal, uninfected mouse brains and their levels of expression increase during parasite infection.

### Expression and distribution of TLR 11–13 proteins among nervous tissue and infiltrating immune cell types

**Figure 2:**
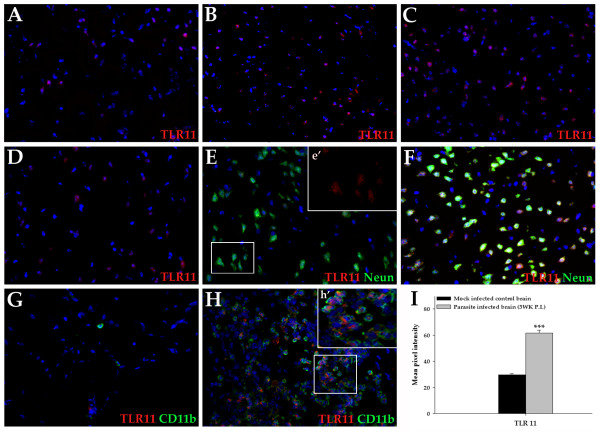
**Expression of TLR11 in brains of mock infected control and NCC mice analyzed by immunofluorescence microscopy**. Balb/c mice were infected intracranially with *M. corti *and sacrificed at various times p.i. Brain crysections were analyzed for expression of TLR11 and cell specific markers using fluorochrome conjugated antibodies. Nuclear staining DAPI is blue. (A) Mock infected control stained with TLR11 specific antibody (400×). (B) *M. corti *infection at 1 wk showing TLR11 positive staining in neurons (400×). (C) *M. corti *infection at 3 wk when maximum TLR11 expression was observed (400×). (D) *M. corti *infection at 6 wk showing TLR11 positive staining (400×). (E) Mock infected control mice stained with TLR11 and neuron specific antibody (Neun) (400×). The insert e' represents a 2× magnification of a selected area depicting TLR11 expression (Red) on neurons. (F) *M. corti *infection at 3 wk showing TLR11 positive neurons, merged image (yellow/orange; 400×). (G) Mock control mice stained with anti-TLR11 and anti-CD11b antibody (400×). (H) *M. corti *infection at 1 wk showing TLR11 and CD11b double positive cells, merged image (yellow/orange; 400×). Insert h' is a 2× magnification of a selected area to better illustrate TLR11 and CD11b double positive staining (I) The relative levels of TLR11 expression in mock and *M. corti *infected animals was calculated as described in experimental procedures.

**Figure 3:**
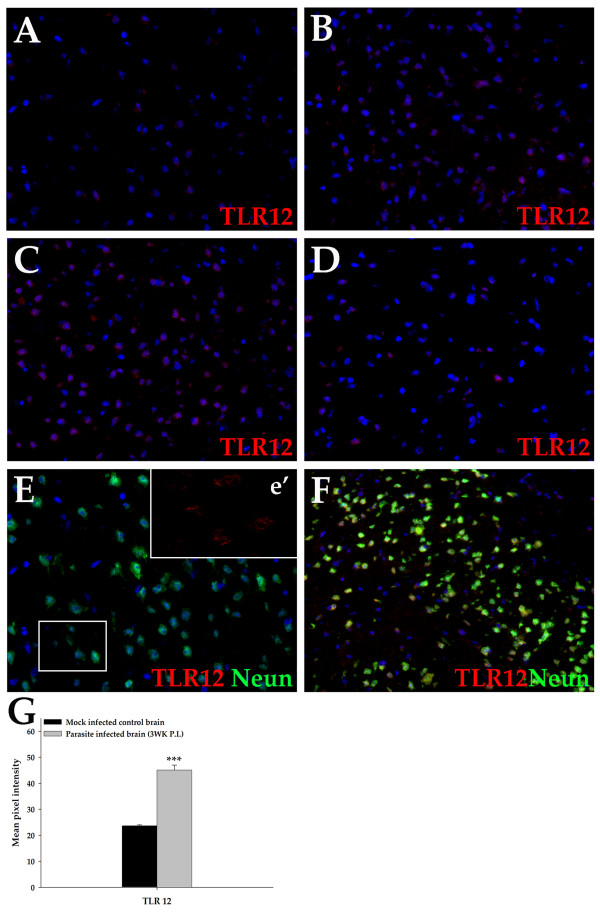
**Expression of TLR12 in brains of mock infected control and NCC mice analyzed by immunofluorescence microscopy**. Balb/c mice were infected intracranially with *M. corti *and sacrificed at various times p.i. Brain cryosections were analyzed for expression of TLR12 and cell specific markers using fluorochrome conjugated antibodies. Nuclear staining DAPI is blue. (A) Mock infected control stained with TLR12 specific antibody (400×). (B) *M. corti *infection at 1 wk showing TLR12 positive staining (400×). (C) *M. corti *infection at 3 wk when maximum TLR12 expression was observed (400×). (D) *M. corti *infection at 6 wk showing TLR12 positive staining (400×). (E) Mock control mice stained with TLR12 and neuron specific antibody (Neun) (400×). The insert e' represents a 2× magnification of a selected area depicting TLR12 expression (Red) on neurons. (F) *M. corti *infection at 3 wk showing TLR12 positive neurons, merged image (yellow/orange; 400×). (G) The relative levels of TLR12 expression in mock and *M. corti *infected animals was calculated as described in experimental procedures.

**Figure 4:**
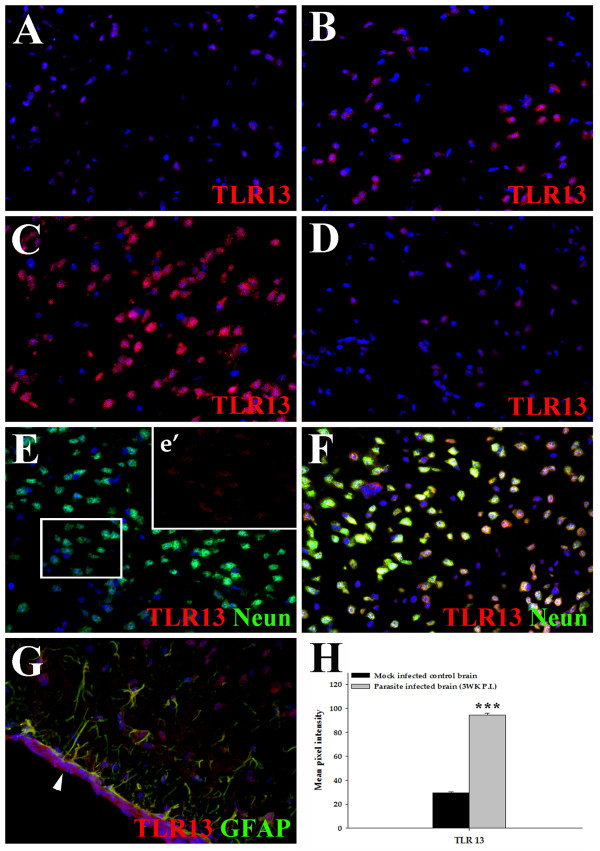
**Expression of TLR13 in brains of mock infected control and NCC mice analyzed by immunofluorescence microscopy**. Balb/c mice were infected intracranially with *M. corti *and sacrificed at various times p.i. Brain cryosections were analyzed for expression of TLR13 and cell specific markers using fluorochrome conjugated antibodies. Nuclear staining DAPI is blue. (A) Mock infected control stained with TLR13 specific antibody (400×). (B) *M. corti *infection at 1 wk showing TLR13 positive staining in neurons (400×). (C) *M. corti *infection at 3 wk when maximum TLR13 expression was observed (400×). (D) *M. corti *infection at 6 wk showing TLR13 positive staining (400×). (E) Mock control mice stained with TLR13 and neuron specific antibody (Neun) (400×). The insert e' represents a 2× magnification of a selected area depicting TLR13 expression (Red) on neurons. (F) *M. corti *infection at 3 wk showing TLR13 positive neurons, merged image (yellow/orange; 400×). (G) *M. corti *infection at 3 wk showing TLR13 positive astrocytes and its processes, merged image (yellow/orange; 400×). The arrow points to the pial vessel expressing TLR13. (H) The relative levels of TLR13 expression in mock and *M. corti *infected animals was calculated as described in experimental procedures.

*In situ *IF microscopy analyses revealed the distribution of TLR 11–13 proteins in brain tissues from mock infected control mice and NCC mice. Brain tissue cells were determined by their characteristic morphology together with cell markers: microglia (CD11b), astrocytes (GFAP), and neurons (NeuN). Surface markers helped identify infiltrating cells: macrophages/microglia cells (CD11b), dendritic cells (CD11c), B-cells (CD19), and αβ T cells (TCR β chain). Brain tissues were analyzed at 1 wk, 3 wk, and 6 wk p.i. At least three mice were analyzed per time point with reproducible results. TLRs 11–13 staining was found to be upregulated in the CNS as a result of infection (Fig. 2, 3, 4).

#### TLR11

TLR11 IF staining was evident in brain tissue cells of both uninfected (Fig. 2A and 2E) and infected mice (Fig. 2B, C, D and 2F), and in infiltrating immune cells during parasite infection (Fig 2H). In the uninfected animals, TLR11 expression was detected at a low basal level mostly in neurons as shown in red color in the insert of a portion of the frame in Fig. 2E (colliculus posterior). The positive staining was present in the frontal cortex, anterior-posterior and external colliculi. In addition, TLR11 expression in neurons was visible in the stratum pyramidale hippocampi (CA1 and CA2), but not in CA3 neurons. At 1 wk p.i., TLR11 protein levels increased in the above mentioned brain areas (Fig. 2B, colliculus posterior), and further increased by 3 wk p.i (Fig. 2C). However, at 6 wk p.i., TLR11 protein levels decreased (Fig. 2D) to levels similar to 1 wk p.i. (Fig. 2B). Morphological, anatomical correlation, and colocalization of TLR11 and Neun staining strongly suggested that the upregulated TLR11 expression was mainly in neurons (Fig. 2F). We quantified protein expression by measuring the mean pixel intensity. Figure 2l shows upregulated TLR11 protein expression in parasite infected brain at 3 wk p.i. Staining, however, was undetected in the nervous tissue cells present in the cerebellum and periventricular and leptomeningeal (LM) areas in either uninfected or in parasite infected NCC mice.

The highest number of infiltrating cells expressed TLR11 in infected brain tissue. Immunofluorescent staining detected no TLR11 infiltrating cells in mock infected mouse brain (Fig. 2G). At 1 wk p.i. about half of the immune cells present in meninges and ventricles were positive for TLR11 protein (Fig. 2H, third ventricle). However, as the length of time p.i. increased, the number of TLR11 positive leukocytes progressively declined. At 3 wk p.i. only a small number of infiltrating cells (~5%) expressed TLR11 protein. At 6 wk p.i., TLR11 positive infiltrating cells were barely present. This is in contrast to the maximal staining observed for TLR11 in nervous tissue cells at 3 wk in NCC mice. Double IF analysis with anti-TLR11 and antibodies to cell surface markers specific for various immune cells indicated that the majority of TLR11 positive cells were CD11b positive myeloid cells (~90%). A few CD11c positive dendritic cells (approximately 5%) were also present (data not shown). However, as microglia also express both CD11b and CD11c, it is possible that some of these TLR11 positive cells detected in the extraparencymal areas of CNS can be migrating microglia as well.

#### TLR12

TLR12 expression was evident in brain tissue cells of both uninfected (Fig. 3A and 3E) and infected mice (Fig. 3B, C, D and 3F). The expression in nervous tissue was generally similar to TLR11 expression with respect to cell types and their location. However, TLR12 protein levels in brain areas as well as the number of positive cells in a particular area appeared to be less than TLR11. TLR12 positive cells in normal or mock-infected animals were fewer in number and displayed only low levels of protein (Fig. 3A &3E). The number of TLR12 positive nervous tissue cells increased at 1 wk p.i. (Fig. 3B). These induced effects were even more pronounced and appeared to reach a maximal expression level at 3 wk p.i. (Fig. 3C and Fig. 3F, yellow/orange). At 6 wk p.i. TLR12 protein levels, measured by both intensity of staining and number of positive cells, substantially decreased (Fig. 3D). Measurement of mean pixel intensity of TLR12 staining confirmed that parasite infection increased expression of TLR12 protein at 3 wk p.i. in the whole brain (Fig. 3G) as compared to the controls.

Similar to TLR11, among nervous tissue cells, TLR12 staining was abundant in neurons (Fig. 3E, red in the insert e' &3F), but not in astrocytes and ependymal cells (data not shown). In addition, TLR12 staining was present in brain areas such as the anterior-posterior and external colliculi and pyramidal neurons of the hippocampus (CA1 and CA2). However, their pattern of expression was different in infiltrating immune cells where only a few immune cells (approximately 5%) were positive for TLR12. The TLR12 positive infiltrating cells were mostly CD11b positive myeloid cells (data not shown). Taken together, the constitutive expression of TLR12 in normal brain and its increased expression during NCC suggest a possible role in CNS function.

#### TLR13

TLR13 expression was evident in brain tissue cells of both uninfected (Fig. 4A and 4E) and infected mice (Fig. 4B, C, D, 4F and 4G). TLR13 staining was predominantly in neuronal cells of both uninfected and infected mice (Fig. 4A–G). In mock infected/normal mouse brain, TLR13 was present in some astrocytes in periventricular areas and in proximity to the meninges (data not shown). However, a much greater number of neurons expressed TLR13 in these brains (Fig. 4A and 4E, red in the insert e'). In normal uninfected animals, a few blood vessels, microglia and ependymal cells were positive for TLR13 (data not shown). The positively stained neurons were in the cerebral cortex adjoining fissura longitudinales, frontal cortex, and anterior-posterior and external colliculi. Astrocytes for TLR13 were solely in the periventricular, and pial and subpial areas. In comparison to TLR11 and 12, TLR13 expression was present in additional areas including periventricular areas, fissure longitudinalis cerebri and CA3 pyramidal neurons of the hippocampus that were negative for TLR11 and TLR12 staining.

Parasite infection produced higher levels of TLR13 protein by several fold (Fig. 4B, C, D, 4F, G and 4H). After reaching maximal expression at 3 wk p.i. (Fig. 3C), levels of TLR13 progressively decreased at 6 wk p.i. (Fig. 4D), but always exhibited elevated levels irrespective of the length of time post infection, even at 10 wk p.i (data not shown). The increased expression of TLR13 at 3 wk p.i. was compared with mock infected mice and expressed as mean pixel intensity (Fig. 4H). TLR13 staining was most evident in nervous tissue cells of infected mice, predominantly in neuronal cells (Fig. 4F). A few infiltrating CD11b myeloid cells were also detected positive for TLR13 (data not shown). In addition to neurons, increased TLR13 expression was present in other CNS cells including astrocytes (Fig 4G), Purkinje cells, ependymal cells and endothelial cells of pial blood vessels (Fig 4G, arrow points to positive pial vessel). A striking increase in TLR13 expression was apparent in terms of both intensity and numbers of astrocytes expressing them. The increased staining in astrocytes was most obvious in periventricular and leptomeningeal white matter areas and in the cerebellar white matter of the brain. In contrast, astrocytes present in parenchymal gray matter areas were not positive for TLR13 staining. The maximum numbers of neurons and astrocytes expressing TLR13 was observed at 3 wk p.i. (Fig. 4G, yellow). Nevertheless, similar to TLR12, TLR13 expression was predominantly in neurons (Fig. 4E and 4F, yellow/orange color in merged image).

## Discussion

Historically, the CNS has been regarded as an immune-privileged organ [35]. However, we now recognize that the CNS possesses active immune processes and control mechanisms [36]. Pattern recognition receptors (PRRs) that constitute the recognition system may be involved in altered brain homeostasis and several CNS diseases with diverse causes such as experimental brain injury caused by stereotactic transection of axons in the entorhinal cortex [37], ischemia [38-42], and autoimmune diseases [43]. Signaling through PRRs appears to elicit innate immune responses that eventually culminate in specific adaptive immune responses [44] and could conceivably generate neuroprotective benefits [27]. In this report we studied the expression and distribution of TLRs 11–13 in the brain under normal physiological conditions and during murine NCC. Previously only one study reported mRNA expression of TLR 13 in the CNS after Semliki Forest Virus (SFV) infection [45]. This is the first report demonstrating the expression of TLRs 11–13 in the CNS following infection.

This study and our previous findings [12] indicate that TLRs 1–13 exhibit differential expression and regulation in normal and NCC mouse brains. TLR2 and TLR13 were expressed at high levels in almost all CNS cell types both in the uninfected and parasite infected brain. However, TLR13 seems to be highly expressed in neurons while TLR2 is more abundant in astrocytes. Nevertheless, high upregulation following infection of both TLR2 and TLR13 in astrocytes and their foot processes that terminate at blood vessels may provide necessary signals for initiation of infiltration of immune cells to the CNS and the subsequent adaptive immune response [46]. This observation could be important in understanding the role of particular TLRs in pathological processes unique to brain tissue, specifically during NCC.

Vertebrate TLRs can be divided into six subfamilies based on the similarity of amino acid composition, extracellular leucine rich repeat (LLR) length and phylogenetic analysis. The TLR2 subfamily includes TLR1, 2, and 6; TLR9 subfamily: TLR7, 8, and 9; TLR11 subfamily: TLR11, 12, and 13 [47]. TLR3, TLR4 and TLR5 subfamilies each consists of a single member [7,47]. Among all the TLRs, very little data detail the functions and mechanisms of action of TLRs 11–13. One major hindrance is the absence of knowledge about their ligands. TLR11 apparently recognizes uropathogenic bacteria [48]. Usually TLRs belonging to a particular family recognize a particular class of ligands, PAMPs, presumably due to selective pressure for specific PAMP recognition. For example, the TLR2 subfamily is specific for lipopeptide, the TLR3 family for dsRNA, the TLR4 family for LPS, the TLR5 family for flagellin, and the TLR7–9 subfamilies for nucleic acid and heme motifs [8,49-52]. A recent report indicated a profilin-like molecule from the protozoan parasite *Toxoplasma gondii *interacts with TLR11 generating a potent interleukin-12 (IL-12) response in murine dendritic cells that is dependent on myeloid differentiation factor 88 [53]. Ligands for the other TLRs suggest that PAMPs for TLRs 11–13 are likely to be protein components. Other studies from our laboratory indicate that *M. corti *metacestodes release carbohydrate containing molecules directly upon invasion whereas other glycoconjugates are secreted throughout the infection process [54-56]. These molecules may potentially act as specific PAMPs that have not yet been described for TLRs. It may be also possible that infections induce cytokines indirectly to stimulate the production of particular TLR proteins [55-57]. In this light, it is of interest to observe that TLRs 11–13 were detected at a lower level at 6 wk p.i. as compared to 1 wk and 3 wk p.i. In the NCC model, acute infection with *M. corti *is associated with a TH1 response early during infection (1–5 wk). However, after 5 wk p.i., a mixed TH1/2 immune responses along with expression of IL-10 are detected [30]. Such anti-inflammatory responses may contribute to the decreased expression of TLRs 11–13 later in infection helping to diminish inflammation induced tissue damage.

TLRs have a major role in host inflammatory responses. Increasing evidence also indicates that TLRs have a major role in several inflammatory CNS pathologies [58-62]. The critical question is whether TLRs have other roles besides inflammation that may be important in diseases and other CNS functions. In this light, constitutive expression and infection-induced upregulation of particular TLR 11–13 proteins in distinct nervous tissue cell types, especially in neurons is of interest. One possibility is that neurons are able to recognize some forms of infection. Previous studies indicated that neurons express TLR2 [12,63] and TLR3 [45,64]. However, in the latter study, unlike glial cells, neuroblastoma cells could not synthesize IFN-α or pro-inflammatory cytokines upon stimulation. Alternatively, or in addition, perhaps activation of neurons, astrocytes and even endothelial cells of blood vessels [27,64-66], via TLRs 11–13 could augment the neuroprotective functions. Indeed, activation of TLR3 is correlated with the production of neuroprotective, angiogenic and migratory factors by astrocytes [24]. TLR signaling is even involved in adult neurogenesis [67,68].

Although the vertebrate TLRs are not fast-evolving genes, TLRs 11–13 are most divergent compared to other TLRs [47] suggesting possible alternate functions other than recognition of PAMPs. Emerging evidence suggests that TLRs recognize several host molecules during pathological conditions. It is reasonable to expect that TLR recognition and interaction with host molecules might participate in normal homeostatic processes as well. Understanding TLR 11–13 functions in specific CNS cell types and the ligands they recognize should provide insight into their roles in the CNS under normal physiological condition as well as during infection.

## Conclusion

In the present study, we showed that TLRs 11–13 are expressed in the CNS and their expression was significantly upregulated during murine NCC. TLR 11–13 proteins were abundantly expressed in neurons of both normal and parasite infected mouse brains. An enhanced understanding of TLR 11–13 functions in specific CNS cell types, particularly in neurons, should provide insight into their role in initiation of immune reactions and deleterious sequelae or perhaps possible neuroprotective roles in chronic infections, particularly in CNS parasitic infection.

## Competing interests

The authors declare that they have no competing interests.

## Authors' contributions

Conceived and designed the experiments: BM JT. Performed the experiments: BM UG. Analyzed the data: BM UG JT. Contributed reagents/materials/analysis tools: JT. Wrote the paper: BM UG JT. All authors have read and approved the final manuscript.
